# Editorial: Recent advancements in RNA technologies, diagnostics, and therapeutics

**DOI:** 10.3389/fbioe.2025.1550225

**Published:** 2025-02-04

**Authors:** Jaimie Marie Stewart, Yaroslava G. Yingling, Kirill A. Afonin, Florent Hubé, Naoyuki Kataoka

**Affiliations:** ^1^ Department of Bioengineering, University of California, Los Angeles, CA, United States; ^2^ Department of Materials Science and Engineering, North Carolina State University, Raleigh, NC, United States; ^3^ Nanoscale Science Program, Department of Chemistry, University of North Carolina Charlotte, Charlotte, NC, United States; ^4^ Laboratoire Biologie du Développement, Institut de Biologie Paris-Seine, Transgenerational Epigenetics and Small RNA Biology, Sorbonne Université, CNRS, Paris, France; ^5^ Laboratory of Cellular Biochemistry, Department of Animal Resource Sciences, Graduate School of Agriculture and Life Sciences, The University of Tokyo, Tokyo, Japan

**Keywords:** RNA technologies, miRNA, circRNA, RNAi, theranostics, tissue engineering, RNA circuits, synthetic biology

## Introduction

RNA technology is an emerging field that exploits the unique structural and functional properties of RNA to build nanoscale structures and regulate complex biological systems (Stewart, 2024). RNA has been shown to assemble into structures with various shapes, sizes, and complexities, enabling applications in molecular sensing, drug delivery, immunomodulation, and cellular activity regulation (Chandler et al., 2021). This foundational work shows the significant potential of RNA molecules and their chemical analogs as a biomaterial for developing personalized diagnostic and therapeutic applications, as evidenced by numerous *in vitro* and *in vivo* studies and exemplified by several FDA approved formulations. However, critical challenges such as nuclease stability, targeted delivery of RNA therapies, regulation of their immune response, and lowering detection limits that must be further addressed to fully translate RNA nanotechnology into clinical applications.

This Research Topic highlights recent advancements and innovative work in RNA technologies for diagnostics and therapeutics of various classes of RNAs. This Research Topic features six review and research articles curated by international leaders in the fields of nucleic acid technologies, drug delivery, and computational studies. All manuscripts present a wide range of innovative technologies encompassing the design and optimization of gene therapies, production of RNAs, logic gating, tissue engineering and verifications of new therapeutic targets.

## MicroRNA for regenerative medicine

Over 30 years ago, the first microRNA (miRNA) was identified in *Caenorhabditis elegans* (Lee et al., 1993; Wightman et al., 1993) and provided insights into how RNA can regulate gene expression. MicroRNAs (miRNAs) are small non-coding RNAs, ∼22 nucleotides in length, that function as key regulators of gene expression. Through their regulatory activity, miRNAs can modulate several biological processes, including cellular differentiation, proliferation, and apoptosis (O'Brien et al., 2018). miRNAs are a promising strategy for advancing regenerative medicine, however, it is imperative to further elucidate miRNA regulatory mechanisms and pathways for clinical applications. Shahin et al. investigated the regulatory role and regenerative functions of microRNA (miR-155) in skin wound repair. The authors performed computational analysis of differentially expressed miRNAs in adipose-derived mesenchymal stem cells (AD-MSCs) and keratinocytes. hsa-miR-155 was identified and experimentally validated as having an enhanced immunomodulatory effect of AD-MSCs through regulating key wound healing proteins FGF2, FGF7, CCL2, and VCAM1. Castañón-Cortés et al. highlighted recent advancements in integrating miRNA into tissue-engineering scaffolds for optimized tissue repair and regeneration. The authors summarize the use of tissue-engineered scaffolds in combination with miRNAs applied to skin, musculoskeletal, nervous, and cardiovascular systems. Additionally, the study emphasizes the need for further experiments to understand fundamental miRNA-mediated mechanisms of action for specific tissue regeneration and minimal off-target effects. Lastly, the current challenges of cellular uptake and localized delivery are outlined.

## Optimizing design approaches for RNA synthesis and gene expression

Engineering molecular machinery and RNA sequences are a practical approach for efficient RNA synthesis and gene regulation capabilities. Circular RNAs (circRNAs) are a highly stable class of non-coding RNAs with diverse biological roles, including acting as miRNA sponges, transcriptional regulation, protein recruitment, and enhancing protein activity (Kristensen et al., 2019). He et al. developed a one-pot process for circRNA synthesis by introducing specific mutations to T7 RNA polymerase (RNAP) to yield a thermostable variant that combines transcription and cyclization in a single reaction. The authors used consensus and folding free energy calculations for hotspot selections to construct a multisite mutant T7 RNAP. The engineered polymerase demonstrated stable activity at 45°C for over an hour, introducing new techniques for efficient circRNA production via a one-pot transcription and cyclization. RNA sequence design is another approach that can be used to tune gene expression. Codons are trinucleotide sequences that determine a specific amino acid. Due to the redundancy of the genetic code, synonymous codons can encode the same amino acid. However, these synonymous codons are not used equally, causing a codon bias (Novoa and de Pouplana, 2012). Paremskaia et al. presented a comprehensive analysis of current metrics for codon optimization for clinical use for gene therapy. The authors categorize methods to generate optimized sequences variants and protocols to experimentally verify mRNA stability and protein expression. The study concludes with persistent challenges of unintended effects on protein function and complexities in evaluating codon effectiveness.

## Synthetic circuits

RNA molecules such as RNA aptamers, ribozymes, and riboswitches can organize to form networks that can perform complex functions including gene regulation, signal amplification, and logic operations for diagnostics and therapeutics (Pfeifer et al., 2023). Tian et al. engineered Boolean logic gates in the yeast *Saccharomyces cerevisiae* by reintroducing the naturally absent RNA interference (RNAi) pathway. The authors found that promoter leakage of pGAL1 inhibited logic behavior. Promoter leakage was reduced when the DNA fragment was placed between the two promoter regions, vastly improving the circuit reliability and performance. Armstrong and Isalan discussed using RNA-based circuits for the development of bacterial theranostics. The authors emphasized the use of mRNA and riboregulatory to construct circuits and program bacteria to sense and respond to physiochemical signals. The study closes with current issues of safety and proving a promising outlook for developing precise and versatile therapeutic systems.

## Outlook

RNA technologies offer innovative approaches for therapeutic and diagnostic applications. However, broad application of RNA-based technologies in clinical settings will require focused research efforts in key areas: increasing target specificity and delivery efficiencies, improving stability and functional retention at ambient temperatures, ensuring precise patient-specific regulation of toxicities, and decreasing production and handling costs. Addressing these critical factors will be essential in realizing the full potential of RNA technologies for widespread clinical use.
